# Infographic. Exercise for intermittent claudication

**DOI:** 10.1136/bjsports-2019-101930

**Published:** 2020-02-12

**Authors:** Garry A Tew, Louise Allen, Christopher D Askew, Ian Chetter, Gabriel Cucato, Patrick Doherty, Andrew Garnham, Amy Harwood, Lee Ingle, Michael Jenkins, Jonathan Michaels, Sara Pittack, Chris Seenan, Hazel Trender

**Affiliations:** 1 Department of Sport, Exercise and Rehabilitation, Northumbria University, Newcastle, UK; 2 Department of Health Sciences, University of York, England, York, UK; 3 Imperial College Healthcare NHS Trust, London, UK; 4 School of Health and Sport Sciences, University of the Sunshine Coast, Maroochydore DC, Queensland, Australia; 5 Sunshine Coast Health Institute, Sunshine Coast University Hospital, Sunshine Coast, Queensland, Australia; 6 Hull York Medical School, Hull, UK; 7 Royal Wolverhampton Hospitals NHS Trust, Wolverhampton, UK; 8 Thermal Ergonomics Laboratory, Discipline of Exercise and Sport Science, The University of Sydney, Sydney, New South Wales, Australia; 9 Department of Sport, Health & Exercise Science, University of Hull, Hull, UK; 10 School of Health and Related Research, The University of Sheffield, Sheffield, UK; 11 Your Thinking Ltd, London, UK; 12 Department of Physiotherapy and Paramedicine, Glasgow Caledonian University, Glasgow, UK; 13 Sheffield Teaching Hospitals NHS Foundation Trust, Sheffield, UK

**Keywords:** exercise training, exercise rehabilitation

**Figure:**
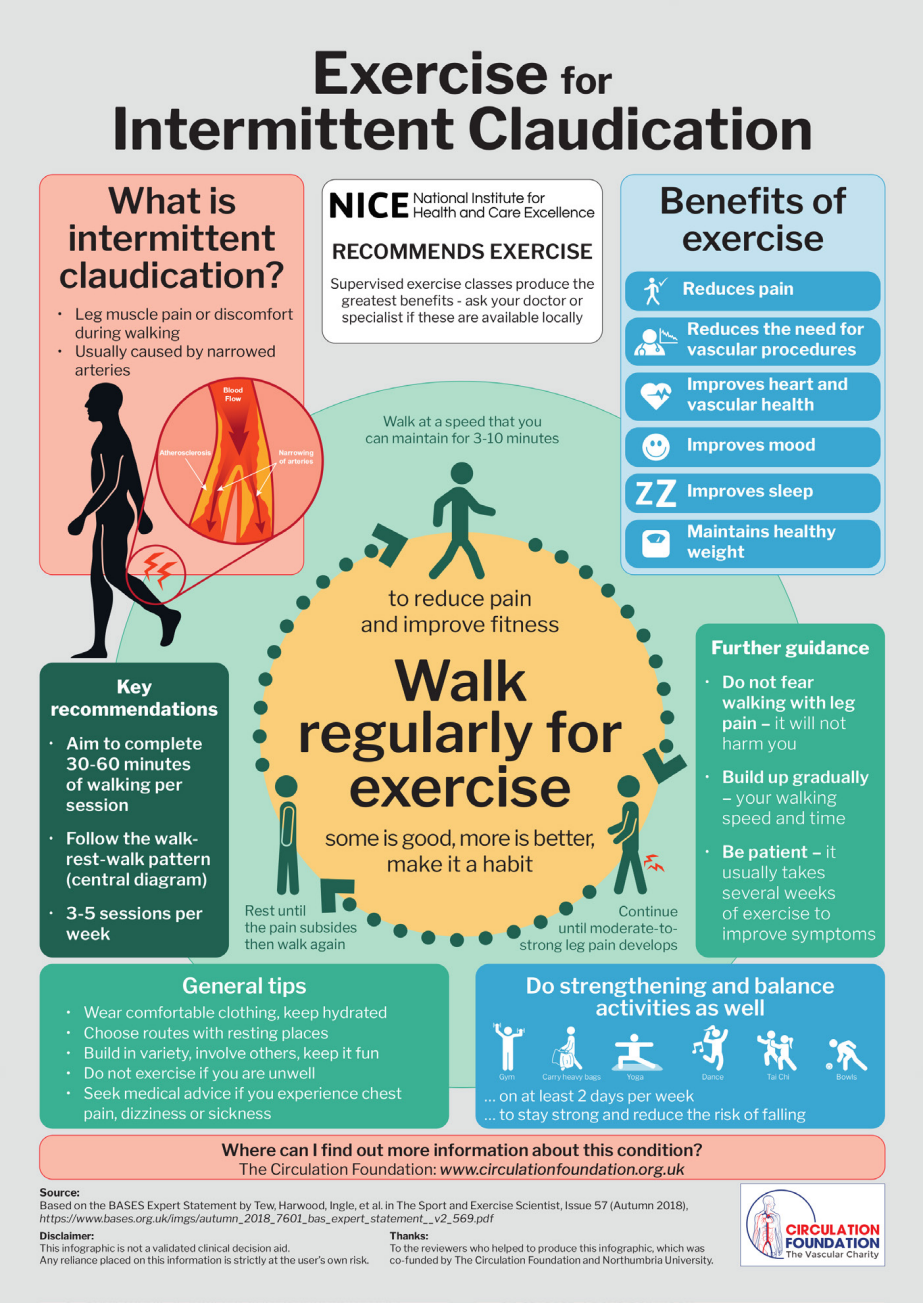


Intermittent claudication (IC) is pain or discomfort in the muscles of the calf, thigh or buttock that occurs during walking and is relieved by rest. It affects 4% of people over 60 years of age and is the most common symptom of peripheral arterial disease (PAD). For people with IC, the goals of treatment are twofold: (1) secondary prevention of cardiovascular disease through management of risk factors (eg, tobacco use, dyslipidaemia, diabetes, hypertension and physical inactivity); (2) improving functional status, with treatment options including exercise training, revascularisation and vasodilator therapy.^1^

In 2012, the UK’s National Institute for Health and Care Excellence published a clinical guideline on the diagnosis and management of PAD.^1^ This guideline recommended that a 3-month supervised exercise programme (SEP) should be offered as a first-line therapy for IC, and that revascularisation and vasodilator therapy should only be considered if exercise provides insufficient symptom relief. Although research studies have shown unsupervised exercise to be generally less effective at improving functional status than an SEP, it can still be effective, and should be recommended if an SEP is not available.^1 2^

The evidence supporting the efficacy of exercise for people with IC dates back to 1966 when a study reported that 6 months of interval walking exercise improved patients’ pain-free and maximum walking distances.^3^ Over the following 50+ years, numerous randomised trials and meta-analyses have been published supporting the efficacy of exercise in improving functional status in this population.^4^ Despite this evidence and the clinical guideline recommendations, the provision of SEPs is variable, with one study reporting that only 38.5% of vascular units in the UK had access to an SEP.^5^ Potential barriers include a lack of funding, facilities and patient motivation.

The benefits of exercise for people with IC are too great to be ignored. Therefore, to support the provision and uptake of exercise, we have developed two new resources. First, a statement for healthcare professionals that summarises the evidence and provides exercise prescription guidelines.^2^ Second, an infographic of key messages aimed primarily at patients. This infographic, which may be shared digitally or used as a poster or handout in clinics, aims to encourage patients to make exercising a regular habit by highlighting potential benefits and providing clear guidelines and safety messages. We hope that readers will share this infographic widely to enhance awareness of this debilitating condition and the important role that exercise can play in its management.
